# Parental satisfaction with care given in neonatal intensive care units, multicentre referral hospitals in eastern Ethiopia

**DOI:** 10.3389/fped.2025.1417869

**Published:** 2025-03-07

**Authors:** Mekdes Workie Dagnew, Aboma Motuma, Arsema Gebreyesus, Kasiye Shiferaw

**Affiliations:** School of Nursing and Midwifery, College of Health and Medical Sciences, Haramaya University, Harar, Ethiopia

**Keywords:** parental satisfaction, associated factors, neonatal intensive care unit, eastern Ethiopia, referral hospital

## Abstract

**Background:**

Parental satisfaction is an important dimension of the quality of a child's care and has been shown to improve health outcomes, including adherence to therapeutic regimens and understanding of medical information. Parental satisfaction is low in resource-limited countries like Ethiopia, with the paucity of evidence. This study aimed to determine the magnitude of parental satisfaction with care given in neonatal intensive care and its associated factors among parents of neonates admitted to neonatal intensive care units (NICUs) at referral hospitals in eastern Ethiopia from June to August 2023.

**Method:**

A facility-based cross-sectional study was conducted among 418 parents whose new-borns were admitted to the NICU and who were selected using a systematic random sampling technique. A pretested semi structured questionnaire was used to collect the data through face-to-face interviews with participants, and a checklist was used to review the charts/records by trained and experienced bachelor nurse/midwife data collectors. The data were coded, entered into Epi-Data version 4.6, and transferred to SPSS version 25 for analysis. We used binary logistic regression analysis to identify factors associated with the outcome variable. An adjusted odds ratio (AOR) with a 95% confidence interval (CI) was used to report the findings, and a *p*-value ≤0.05 was considered to indicate statistical significance.

**Results:**

The magnitude of parental satisfaction with care given in the NICU was 50.5% (95% CI: 45.6–55.5). Being a rural resident (AOR = 2.13; 95% CI: 1.33–3.43), having a shorter hospital stay (AOR = 4.25, 95% CI: 2.08–8.69), being able to breastfeed (AOR = 2.46; 95% CI: 1.48–4.09), having a single birth (AOR = 4.16; 95% CI: 1.91–9.03), and the availability and quality of the family room (AOR = 2.36; 95% CI:1.40–3.99) were significantly independent associated factors with parental satisfaction with care given in the NICU.

**Conclusion:**

Only one in two parents were satisfied with the care given in the NICU. The present study highlights that shortening hospital stays, mothers' ability to breastfeed their newborns, having a single birth, and the availability and quality of the family room contribute to enhancing parental satisfaction with care given in the NICU in eastern Ethiopia.

## Introduction

Parental satisfaction is the balance between a parent's expectations of the ideal care to be given and the observation of the real and available care given to their neonate (1). Having an infant in the NICU is a stressful and painful experience for the parents of newborns. Similarly, parents dissatisfied with the care given in the NICU faced increased anxiety and a lack of confidence in the health system (2). Moreover, lack of parental satisfaction makes the parents apprehensive, afraid to inquire about their child, and possibly unable to properly care for the child, which leads to deficient neonatal problem detection and management (3), parents who choose to discontinue medical treatment against the advice of medical professionals and who prevent their children from using health services again present serious obstacles to the success of universal health coverage. As a result, there is an increase in neonatal mortality, morbidity, and readmission rates (4–6). Further neonatal mortality results from the poor quality of neonatal care services in a health care setting particularly, parents not satisfied with care given at the NICU (7).

Studies across low-income countries revealed significant contributors to parental satisfaction were length of hospital stay (8), residence, educational status, clear explanation about procedures and medications, birth weight of baby, and allowing visitors (9–11). Since parent satisfaction with neonatal care is an indicator of good quality care, its recognition is a vital step for the reduction and control of neonatal morbidity and mortality (12) in which parents become a central part of the NICU care team like Kangaroo Mother Care (KMC), resulting in greater neonatal weight gain, higher rates of exclusive breastfeeding at discharge, decreased hospital readmission, and lower parental stress levels (13).

Although different studies have been conducted in our country, each of the studies was conducted at a single health facility; as a result, we conducted this study in a wide geographical area to make it more representative of the population of the study and improve generalizability. Moreover, factors such as the number of current births, mode of delivery, and baby's ability to breastfeed, which affect parental satisfaction with NICU care, were not addressed in previous studies.

The purpose of this study was primarily to help the three referral hospitals detect areas that need improvement, specifically parent participation or involvement in the NICU to initiate family-centred care, and to help healthcare providers offer appropriate and excellent health care by identifying the factors that affect parent satisfaction by involving the parents as part of the team in caring for the neonate in the NICU and providing a good basis for future researchers. Therefore, this study aimed to identify the magnitude of parental satisfaction and the factors associated with care given in the NICU at referral hospitals in eastern Ethiopia.

## Materials and methods

### Study setting, period, and design

All referral hospitals which were located in eastern Ethiopia such as Hiwot Fana Comprehensive Specialized Hospital, Dil Chora Referral Hospital, and Sheik Hassan Yabere Referral Hospital were included in this study. The study was conducted at three referral hospitals in eastern Ethiopia from June 15 to August 15, 2023. A facility-based cross-sectional study design was conducted.

### Population

All parents whose neonates were admitted to the NICU at referral hospitals in eastern Ethiopia composed the source population, whereas parents whose neonates were admitted to the NICU at referral hospitals in eastern Ethiopia who were available during the study period composed the study population.

### Inclusion and exclusion criteria

All parents whose neonates completed their care and treatment from the neonatal intensive care unit and prepared for discharge were included, and parents of neonates who unable to communicate due to mental or physical conditions were excluded.

### Sample size determination and sampling procedure

The sample size was calculated based on a single population proportion formula with the following assumptions: 95% confidence level, 5% margin of error, and proportion = 0.55 from a previous study (11).

$$n=(Za/2{)}^{2}{\;p(1-P)/\left(d\right)}^{2}$$

where *n* = the sample size and *Z* = the standard normal distribution curve at the 95% confidence level, with a value of 1.96.

*P* = previous proportion of satisfaction in NICU care, *d* = the level of precision between sample and population (degree of accuracy desired).

Then, *n* = (1.96)^2^ (0.55) (1–0.55)/(0.05)^2^ = 380, and after adding 10% nonresponses, the final sample size was 418.

The sampling procedure was performed, first by calculating the source population from 2-month neonatal admission reports from the previous year in a similar month taken from each referral hospital obtained from their health management information system data. Since the data were collected for 2 months because of low patient flow. 280 in Hiwot Fana Comprehensive Specialized Hospital and 240 in Dil Chora Referral Hospital and 320 Sheik Hassan Yabare Referral Hospital have average neonatal admission. There were a total of 840 neonatal admission in all three hospitals, which was taken as a source population to calculate the proportional sample size of the respective individual hospital by using the formula of *ni* = n*Ni/*N*. Then, the calculated sample size (418) was proportionally allocated to each hospital to determine the required number of participants. Then, a systematic random sampling technique was used to recruit every two eligible participants from the NICU by using their neonatal registration numbers from the neonatal admission logbook.

### Data collection methods

The questionnaire was adapted a validated tool called the Empowerment of Parents in the Intensive Care-Neonatology (EMPATHIC-N) (14). Formerly, it was developed in the Netherlands with 57 items and five domains, and the study performed in Ethiopia was validated and reduced to 38 items. The scores of parents' satisfaction with neonatal intensive care ranged from a minimum of 38 to a maximum of 190 out of the 38 satisfaction-measuring questions on a 5-point Likert scale.

The level of satisfaction for each statement in the questionnaire ranged from 1 to 5, with 1 indicating the lowest level of satisfaction (very dissatisfied) and 5 indicating the highest level of satisfaction (very satisfied). The data collectors were BSc midwives who did not work in the study area and were supervised by the principal investigator and four MSc midwives.

The data were collected using a pre-tested semi structured questionnaire through interviewer-administered face-to-face interviews and chart reviews for neonatal-related questions. The data collectors were responsible for interviewing the parents in the private room immediately following the discharge of the new born from the NICU, and the supervisors were assured that the data collection process was planned.

### Variables and measurements

Parental satisfaction status (satisfied or dissatisfied) was the outcome variable. The independent variables included age (≤24, 25–34, ≥35), educational status (unable to read and write, primary school, grade 9–12, certificate/diploma, degree and above), residence (urban, rural), marital status (married, divorced, windowed), parental sex (male, female), and parental occupation (governmental, farmer, merchant, housewife, other).

Further explanatory variables included length of hospital stay (≤7, 8–14, ≥15 days), gestational age of newborn (term, preterm, post-term), history of admission (none, once and more), admission diagnosis (prematurity, infection, jaundice, birth asphyxia, congenital malformation and others), birth weight (low, normal, macrosomia), breastfeeding status (yes, no), place of delivery (hospital, health center, home), mode of delivery (SVD, C/S, ID), number of current births (single, multiple), availability and quality of family room (satisfied, neutral, dissatisfied), distance between the NICU and postnatal ward (satisfied, neutral, dissatisfied), functionality and cleanness of the toilet (satisfied, neutral, dissatisfied), being asked for consent (satisfied, neutral, dissatisfied), having visors (satisfied, neutral, dissatisfied), and accessibility of enough chairs in the waiting area (satisfied, neutral, dissatisfied).

### Operational definitions

Parents: mothers, fathers, or any designated primary caregivers whose neonates were admitted to the NCU in this study.

Parental satisfaction: The overall satisfaction of parents was measured based on the mean of 38 satisfaction measurement questions (14).

Parental satisfaction: Overall satisfaction was calculated by summing all items measuring satisfaction and was determined by the cut-off point, which is the mean score. Parents who scored equal to or above the mean score were classified as satisfied; otherwise, they were classified as unsatisfied (6, 15).

### Data processing and analysis

The collected information was manually checked for completeness and consistency, and the data were cleaned, coded, entered into Epi-data version 4.6 software, and exported to SPSS version 25 software for further cleaning and analysis. Incomplete questionnaires were excluded from the final analysis and considered nonresponses. Descriptive statistics were carried out to compute the frequency, mean, and standard deviation. The reliability of the items of the EMPATHIC-N tool was checked, and the results showed good internal consistency, with a Cronbach's alpha of 0.977. Finally, the means were calculated; those who scored at least above the mean were considered satisfied, and those who scored below the mean were considered dissatisfied.

After categorizing the overall mean parental satisfaction score, independent variables were analysed using binary logistic regression with parental satisfaction with NICU care. Multicollinearity was checked [the values of the variance inflation factor (VIF)] ranged between 1.083 and 1.658. The Hosmer-Lemeshow goodness of fit test was carried out and confirmed that the analysis model was appropriate, with a *p*-value of 0.702. Variables from the bivariable logistic regression with a *p*-value less than 0.25 were fitted to a multivariable logistic regression. Both the crude odds ratio (COR) in bivariable logistic regression and the adjusted odds ratio (AOR) in multivariable logistic regression with the corresponding 95% confidence intervals were calculated to show the strength of the associations. In the multivariable binary logistic regression analysis, variables with a *p*-value ≤0.05 were considered to be statistically significant.

## Results

### Socio-demographic characteristics of the respondents

Of 418 participants, 408 participated in the study, with a response rate of 97.6%. The mean age of the participants was 29.34 (±5.7 standard deviations, SD) years. Most of the participants (85.5%) were females, whereas more than half of the participants, 225 (55.1%) were from urban areas. The majority of participants, 359 (88%) were married (Table 1).

**Table 1:** Sociodemographic characteristics of parents whose neonates were admitted to the NICU at referral hospitals in eastern Ethiopia, 2023 (*n* = 408).

Variables	Category	Frequency (*n*)	Percent (%)
Sex of respondent	Male	59	14.46
	Female	349	85.54
Age	≤24	94	23.04
	25–34	198	48.53
	≥35	116	28.43
Residence	Urban	225	55.15
	Rural	183	44.85
Occupation	Governmental	75	18.38
	Farmer	46	11.27
	Merchant	105	25.74
	Housewife	125	30.64
	Others	57	13.97
Current marital status	Married	359	87.99
	Divorced	26	6.37
	Windowed	23	5.64
Educational status	Unable to read and write	101	24.75
	Primary school	88	21.57
	Grade 9–12	52	12.75
	Certificate/diploma	95	23.28
	Degree and above	72	17.65

### Neonatal-related characteristics

Two hundred twenty-one (54.2%) neonates were females. Approximately 49.5% of the newborns were preterm, and only 4.7% were post-term at birth. The major contributors to neonatal admission to the NICU were neonatal infection (25.0%), followed by prematurity (15.7%). A total of 57.2% of the newborns had a normal birth weight, and 3.5% were overweight at birth. The duration of hospital stay ranged between 2 and 35 days, with an average of 12.16 (±7.1 SD) days (Table 2).

**Table 2:** Characteristics of neonates who were admitted to the NICU at referral hospitals in eastern Ethiopia, 2023 (*n* = 408).

Variables	Category	Frequency (*n*)	Percent (%)
Sex of neonate	Male	187	45.83
	Female	221	54.17
Length of hospital stay	≤7 days	143	35.05
	8–14 days	137	33.58
	≥15 days	128	31.37
Previous neonate admission	None	318	77.94
	Once and more	90	22.06
Baby breast feeding	Yes	280	68.63
	No	128	31.37
Gestational age	Preterm	161	39.46
	Term	226	55.39
	Postterm	21	5.15
Birth weight	LBW	155	37.99
	Normal birth weight	217	53.92
	Macrosomia	33	8.089
Admission diagnosis	Prematurity	64	15.68
	Infection	102	25.00
	Jaundice	47	11.52
	Birth asphyxia	53	12.99
	Congenital malformation	15	3.68
	Respiratory problem	53	12.99
	Low birth weight	17	4.17
	Hypothermia	30	7.35
	Others	27	6.62

Others included macrosomia, birth trauma, anaemia, neonatal seizure, meconium aspiration syndrome, and haemorrhagic diseases of the newborn.

### Pregnancy-related characteristics

Although six out of seven (85.8%) mothers had initiated ANC follow-up, only 47.30% had attended four or more ANC visits. Approximately 251 (61.5%) of the neonates were delivered via spontaneous vaginal delivery, while 121 (29.7%) of the neonates were delivered via cesarean section. Three hundred fifty-eight (87.75%) of the women had singleton births, and 255 (62.5%) of the neonates delivered at the hospitals (Table 3).

**Table 3:** Obstetric characteristics of parents whose neonates were admitted to the NICU at referral hospitals in eastern Ethiopia, 2023 (*n* = 408).

Variables	Category	Frequency (*n*)	Percent (%)
ANC visit	Yes	350	85.78
	No	58	14.22
Number of ANC visit	Less than four	185	52.71
	Four and more	16	47.29
Mode of delivery	CS	121	29.66
	SVD	256	62.75
	Instrumental	31	7.60
Numbers of current birth	Single	358	87.75
	Multiple	50	12.25
Place of delivery	Hospital	255	62.50
	Health center	132	32.35
	Home	21	5.15

CS, caesarean section; SVD, spontaneous vaginal delivery.

### Hospital-related characteristics

A total of 166 (40.7%) of the participants were satisfied, and approximately 87 (21.3%) of the parents felt neutral about the functionality and cleanliness of the toilets of the hospitals. Approximately 188 (46.1%) and 149 (36.5%) of the parents were dissatisfied and satisfied with the availability and quality of the family room in the ward, respectively (Table 4).

**Table 4:** Hospital characteristics of parents whose neonates were admitted to the NICU at referral hospitals in eastern Ethiopia, 2023 (*n* = 408).

Variables	Category	Frequency (*n*)	Percent (%)
Functionality and cleanness of toilet	Satisfied	166	40.69
	Dissatisfied	155	37.99
	Neutral	87	21.32
Accessibility of enough chairs in the waiting area	Satisfied	161	39.46
	Dissatisfied	187	45.83
	Neutral	60	14.71
Availability and quality of family room	Satisfied	149	36.52
	Dissatisfied	188	46.07
	Neutral	71	17.41
Being allowed to have visitors	Satisfied	160	39.22
	Dissatisfied	189	46.32
	Neutral	59	14.46
Clear explanation about procedures & medication	Satisfied	168	41.17
	Dissatisfied	180	44.12
	Neutral	60	14.71
Easily accessible of unit from postnatal ward	Satisfied	161	39.46
	Dissatisfied	186	45.83
	Neutral	61	14.71
Consent before any clinical procedure	Satisfied	126	30.88
	Dissatisfied	180	44.12
	Neutral	102	25.00

### Magnitude of parental satisfaction

**Figure 1:**
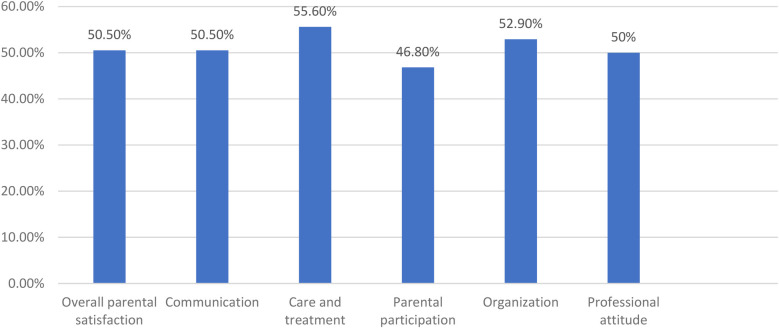
Parents’ overall and dimensional satisfaction with neonates care in NICU at referral hospitals in eastern Ethiopia, 2023 (*n* = 408).

A total of 206 (50.5%; 95% CI = 45.6–55.5) parents were satisfied with the care given in the neonatal intensive care unit at referral hospitals in eastern Ethiopia. Among the domains used to measure the overall satisfaction of parents with NICU care, and treatment-related satisfaction 227 (55.6%) and parental participation-related satisfaction 191 (46.8%) were among the dimensions with the highest and lowest satisfaction scores, respectively (Figure 1).

### Factors associated with the satisfaction of parents with neonates care in NICU

According to the bivariable analysis, residence, birth weight, length of hospital stay, having infants who were able to breastfeed, mode of delivery, gestational age of the neonate at birth, number of current births, availability and quality of the family room and being allowed to have visitors were candidates for the final model, with *p* values ≤0.25. However, multivariable binary logistic regression analysis indicated that place of residence, length of hospital stay, having infants who were able to breastfeed, number of current births, availability and quality of family room were factors significantly associated with parental satisfaction with neonatal care in the NICU (*p* ≤ 0.05).

The odds of parent satisfaction with neonates care in NICU were 2.13 times greater among rural parents than urban dweller parents (AOR = 2.13, 95% CI = 1.33–3.43). Parents who stayed for less than 7 days had 4.25 times greater odds of being satisfied with neonates care in NICU than those who stayed for 15 or more days in the hospital (AOR = 4.25, 95% CI = 2.08–8.69).

The odds of parent satisfaction were 2.46 times greater among parents whose infants were able to breastfeed than among those whose infants were not breastfed (AOR = 2.46, 95% CI = 1.48–4.09). Moreover, parents who had a single birth had 4.16 times greater odds of being satisfied with neonates care in NICU than parents who had multiple births (AOR = 4.16, 95% CI = 1.91–9.03). Those who were satisfied with the availability and quality of the family room were 2.36 times more likely to be satisfied with the services given in the NICU than those who were not satisfied (AOR = 2.36, 95% CI = 1.40–3.99; Table 5).

**Table 5:** Crude and adjusted binary logistic regression analysis for factors associated with parental satisfaction with neonates care in NICU at referral hospitals in eastern Ethiopia, 2023 (*n* = 408).

Variable	Satisfaction		Crude OR (95% CI)	Adjusted OR (95% CI)	*p* value
	Satisfied *N* (%)	Dissatisfied *N* (%)			
Residence					
Rural	116 (63.4)	67 (36.6)	2.60 (1.74–3.88)	2.13 (1.33–3.43)	**0.002**
Urban	90 (40)	135 (60)	1	1	
Length of hospital stay					
≤7 days	107 (74.8)	36 (25.2)	5.7 (3.36–9.59)	4.25 (2.08–8.69)	**0.000**
8–14 days	55 (40.1)	82 (59.9)	1.28 (0.78–2.11)	1.56 (0.87–2.81)	0.136
≥15 days	44 (34.4)	84 (65.5)	1		
Gestational age					
Preterm	58 (36.0)	103 (64.0)	1	1	
Term	134 (59.3)	92 (40.7)	2.59 (1.70–3.92)	0.89 (0.47–1.69)	0.727
Post term	14 (66.7)	7 (33.3)	3.55 (1.36–9.30)	2.27 (0.64–8.05)	0.202
Birth weight					
LBW	53 (34.2)	102 (65.8)	1	1	
Normal birth weight	136 (62.7)	81 (37.3)	3.23 (2.10–4.97)	1.08 (0.547–2.12)	0.830
Macrosomia	17 (51.5)	16 (48.5)	2.04 (0.95–4.37)	0.97 (0.35–2.71)	0.954
Breast feeding					
Yes	165 (58.9)	115 (41.1)	3.04 (1.96–4.73)	2.46 (1.48–4.09)	**0.001**
No	41 (32.0)	87 (68.0)	1	1	
Mode of delivery					
CS	47 (38.8)	74 (61.2)	1	1	
SVD	146 (57.0)	110 (43.0)	2.09 (1.34–3.25)	1.57 (0.93–2.66)	0.094
Instrumental	13 (41.9)	18 (58.1)	1.14 (0.51–2.53)	1.09 (0.43–2.75)	0.657
Numbers current birth					
Single	193 (53.9)	165 (46.1)	3.33 (1.71–6.48)	4.16 (1.91–9.03)	**0.000**
Multiple	13 (26.0)	37 (74.0)	1	1	
Availability and quality of family room					
Satisfied	106 (71.1)	43 (28.9)	3.63 (2.30–5.74)	2.36 (1.40–3.99)	**0.001**
Dissatisfied	76 (40.4)	112 (59.6)	1	1	
Neutral	24 (38.8)	47 (66.2)	0.75 (0.42–1.33)	0.65 (0.33–1.25)	0.198
Being allowed to have visitors					
Satisfied	92 (57.9)	67 (42.1)	1.83 (1.19–2.80)	1.32 (0.79–2.19)	0.282
Dissatisfied	81 (42.9)	108 (57.1)	1	1	
Neutral	33 (55.0)	27 (45.0)	1.63 (0.91–2.92)	1.24 (0.62–2.51)	0.541

Bold: statistically significant at *p* value <0.05; COR, crude odds ratio; AOR, adjusted odds ratio; CI, confidence interval; CS, cesarean section; SVD, spontaneous vaginal delivery; LBW, low birth weight; 1, reference.

## Discussion

This study revealed parental satisfaction with the care given in neonatal intensive care units and associated factors at referral hospitals in eastern Ethiopia. Thus, approximately half of the parents were satisfied with the care given in the intensive care units, but the other half may have concerns or issues with the care their child received; place of residence, duration of hospital stay, infant breastfeeding, number of births, and availability and quality of room for family were significant predictors of satisfaction with the care given in the intensive care units of referral hospitals in eastern Ethiopia.

Overall, 50.5% of the parents were satisfied with the care given in the neonatal intensive care unit, which was consistent with the findings of studies by Debre Tabor (47.8%) (15), Gondar (50%) (10), and Bahir Dar (55.5%) (11). However, this finding is lower than those of studies carried out in Rwanda (66%) and in Ethiopia, such as in Debre Brihan (77%) (9), Jimma (57.9%) (6), and southern Ethiopia (63%) (16). This variation might be due to differences in hospital setups because some of the setups could be well equipped, and skilled manpower and convenient sampling methods were used in some studies (i.e., in Rwanda) (17). In this study, the EPHATHIC tool was used, whereas the NSS-8 tool was used in the Rwanda, Debre Brihan, and Southern studies. A study in Jimma revealed that environmental factors had no significant impact on parental satisfaction, and participants were conveniently selected.

The current magnitude is greater than that in studies conducted in Addis Ababa (41.8%) (18). This discrepancy might be due to studies conducted at different periods, during which there could have been changes in care practices or improvements in the healthcare system. These changes could affect parental satisfaction levels. In addition, most of the respondents in Addis Ababa were urban and relatively literate, which was found to be a significant factor for the decrease in satisfaction, as identified by this study and other previous studies (15).

Parents from rural areas had approximately two times greater satisfaction with neonates care in NICU than those from urban areas, which is consistent with a study conducted in Greece (19) and DebreTabor, Ethiopia (15). The reason could be parents from rural areas may have lower expectations and demands for neonates care in NICU due to limited exposure to advanced medical facilities and technology. Therefore, when they receive care in a NICU, they may perceive as it is good, and be more satisfied compared to parents from urban areas, who might have higher expectations due to greater exposure to advanced medical facilities (20).

Parent satisfaction with neonates care in NICU was four times greater for parents who stayed in the hospital for less than 7 days than for those who stayed in the hospital for 15 or more days, which is consistent with the findings of studies in Gondar (10), Bahrdar (11) and Debretabor (15) Ethiopia. This might be because hospital stays can be expensive, and a shorter stay may result in lower medical costs for parents. This financial relief can contribute to their satisfaction with the NICU care they receive. In addition, a shorter hospital stay may indicate that the neonate is recovered and requires less intensive medical care. This can reassure parents about their child's health and development, leading to higher satisfaction levels with the neonates care in NICU provided (21).

The odds of parent satisfaction were greater among parents whose infants were able to breastfeed than among those whose infants were not able to breastfeed. This finding was also supported by a study conducted in Greece (22). This might be because breastfeeding often requires active participation from parents. They may be involved in latching, feeding routines, and overall care for their baby. This involvement can give parents a sense of control and empowerment in their child's care, leading to increased satisfaction with the NICU experience (23).

In addition, the odds of parent satisfaction with neonates care in NICU among parents who had a single birth were approximately four times greater than those among parents who had multiple births. This is consistent with studies conducted in Greece (22). Parents with a single birth may have more time available to spend with their baby in the NICU than parents with multiple births. This additional time spent with their baby can strengthen the parent-child bond and contribute to higher levels of satisfaction (24).

In this study, parents who were satisfied with the availability and quality of the family room were 2.36 times more likely to be satisfied with the care given in the NICU than those who were not satisfied. This finding is supported by a study conducted in Europe (25). This might be because being satisfied with the availability and quality of a family room can help alleviate some of the stress and anxiety experienced by parents in the NICU. It provides a space where parents can relax, take breaks, and recharge, which can positively impact their well-being. When parents are less stressed, they may perceive NICU care as more satisfactory (21).

### Strengths and limitations of the study

This study covered wide geographical areas and included three hospitals in eastern Ethiopia, which assures generalizability and representativeness. A standard validated tool was used to measure the outcome variable. Moreover, this study addressed factors of ability to breastfeed, the number of current births, and the mode of delivery, which were not addressed by previous studies. However, our study has several limitations. There might be a possibility of recall and social desirability bias during the interviews.

## Conclusion

Only half of the parents were satisfied with the care given in the NICU, which is low compared to other studies. The present study highlights that shortening hospital stays, mothers' ability to breastfeed their newborns, having a single birth, and the availability and quality of the family room contribute to enhancing parental satisfaction with care given in the NICU in eastern Ethiopia.

## Data Availability

The original contributions presented in the study are included in the article/Supplementary Material, further inquiries can be directed to the corresponding author.
